# Posterior Lamellar Reconstruction Using a Free Tarsoconjunctival Graft for a Large Lower Eyelid Defect

**DOI:** 10.7759/cureus.102550

**Published:** 2026-01-29

**Authors:** Aoi Ogura, Yuto Yamamura, Kazuyasu Fujii, Kazutoshi Nishimura, Shunya Usui, Chisa Nakashima, Atsushi Otsuka

**Affiliations:** 1 Department of Dermatology, Kindai University Hospital, Osaka, JPN

**Keywords:** basal cell carcinoma, dermatosurgery, free tarsoconjunctival graft, lower eyelid reconstruction, posterior lamella

## Abstract

Reconstruction of the posterior lamella after excision of lower eyelid malignancies is essential to prevent postoperative functional complications. Free tarsoconjunctival grafting (FTG) allows single-stage reconstruction but is commonly applied to small to moderate defects, as graft survival depends on rapid revascularization. A case of basal cell carcinoma (BCC) of the lower eyelid is presented, in which posterior lamellar reconstruction was successfully performed using FTG for a relatively large defect measuring approximately 15 mm in width, which exceeds the size commonly selected in dermatologic practice. After full-thickness excision with a 2-mm margin, FTG was combined with a cheek rotation flap for anterior lamellar reconstruction. At six months postoperatively, mild ectropion was observed without corneal exposure or lagophthalmos, and both functional and cosmetic outcomes were satisfactory. This case suggests that FTG, when combined with a well-vascularized anterior lamellar flap, may be feasible in selected cases with larger posterior lamellar defects.

## Introduction

Basal cell carcinoma (BCC) and other cutaneous malignancies frequently arise in the lower eyelid, accounting for approximately 5-10% of all skin cancers [1]. The lower eyelid is characterized by thin skin and complex anatomy and plays a critical role in ocular surface protection and tear drainage [2,3]. Consequently, surgical treatment of lower eyelid malignancies requires not only reliable oncologic control but also meticulous reconstruction to preserve eyelid function and achieve satisfactory cosmetic outcomes [4]. When tumor excision results in loss of the posterior lamella, functional complications such as ectropion, corneal exposure, and lagophthalmos may occur; therefore, reconstruction of the posterior lamella is of particular importance [4].

Free tarsoconjunctival grafting (FTG), which utilizes autologous tarsoconjunctival tissue, offers excellent anatomical and functional compatibility and allows for single-stage reconstruction. Accordingly, it has been widely used for posterior lamellar reconstruction [5,6]. However, FTG is a free graft in which the vascular supply is completely interrupted at harvest, and graft survival depends largely on rapid revascularization from the recipient bed. As a result, larger grafts are associated with an increased risk of complications such as partial necrosis and postoperative contraction [7], and FTG is generally considered suitable only for small to moderate defects. In dermatologic series, FTG has generally been applied to small to moderate posterior lamellar defects, with anticipated defect widths of approximately 12 mm, reflecting a cautious approach to graft size selection in this field [8].

Herein, we report a case in which posterior lamellar reconstruction using FTG was successfully performed for a large lower eyelid defect measuring 15 mm in width, achieving favorable functional and cosmetic outcomes.

## Case presentation

An 81-year-old woman presented with a papule with a black dot on the left lower eyelid that had been present for approximately one year. She had no significant medical comorbidities and was not receiving immunosuppressive therapy at presentation. She initially consulted a local dermatologist and was subsequently referred to our department for further evaluation and treatment. Based on the clinical findings, the lesion was diagnosed as BCC. The tumor measured 11 × 6 mm.

**Figure 1:**
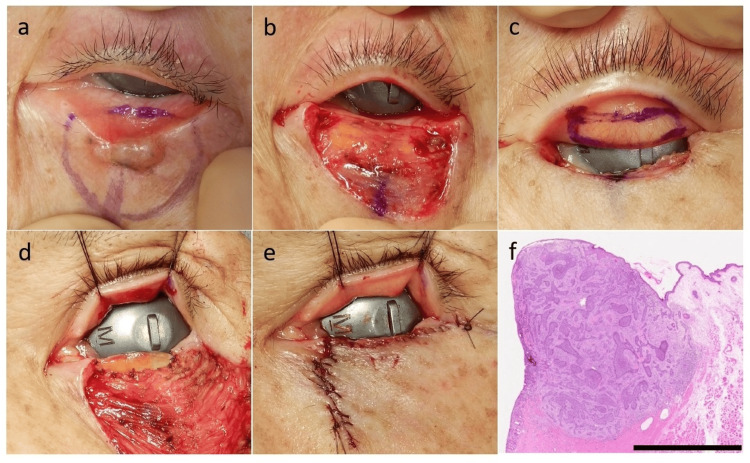
Clinical, intraoperative, and histopathological findings. a: preoperative clinical view showing a papule with a black dot on the left lower eyelid; b: intraoperative view after full-thickness excision of the tumor with a 2-mm surgical margin, including the tarsus and palpebral conjunctiva, resulting in a posterior lamellar defect; c: harvesting of a 15 × 3 mm FTG from the upper eyelid; d: fixation of the FTG to the posterior lamellar defect; e: completion of anterior lamellar reconstruction using a cheek rotation flap; f: histopathological findings demonstrating nodular basal cell carcinoma (hematoxylin and eosin stain). The surgical margins were negative. Scale bar = 2.5 mm. FTG: free tarsoconjunctival grafting

The tumor was excised with a 2-mm surgical margin, including full-thickness resection of the eyelid involving the tarsus and palpebral conjunctiva (Figure 1a). The resulting posterior lamellar defect measured approximately 15 × 3 mm (Figure 1b). A tarsoconjunctival graft was harvested from the upper eyelid (Figure 1c) and sutured to the posterior lamellar defect (Figure 1d). The donor site on the upper eyelid was left to heal by secondary intention. Reconstruction of the anterior lamella was performed using a cheek rotation flap (Figure 1e). Histopathological examination revealed nodular BCC, and all surgical margins were negative (Figure 1f). The surgical wound epithelialized within approximately three weeks, and the flap survived without complications.

**Figure 2:**
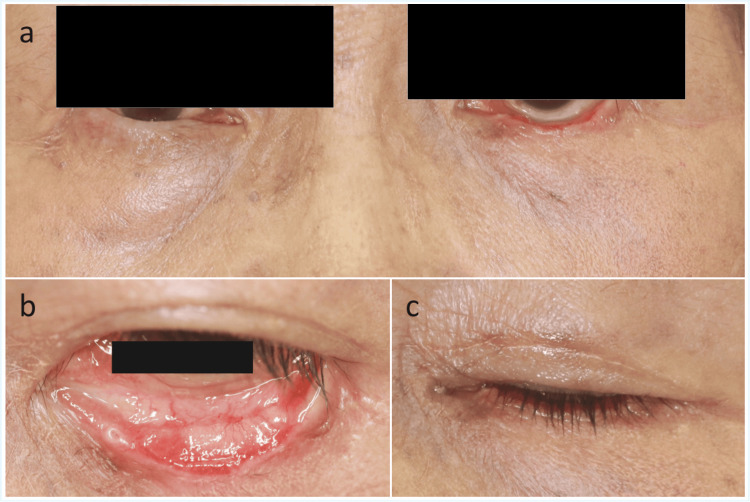
Postoperative findings at six months. a: frontal view showing mild ectropion of the left lower eyelid, with minimal asymmetry between the two sides; b: everted view of the lower eyelid demonstrating good graft integration at the reconstructed posterior lamella, without evident contracture or scar-related changes; c: closed-eye view showing no evidence of lagophthalmos or corneal exposure.

At the six-month postoperative follow-up, mild ectropion was observed; however, there was no evidence of corneal exposure or lagophthalmos. The graft was well-positioned, and both functional and cosmetic outcomes were satisfactory (Figures 2a-2c). No tumor recurrence was observed.

## Discussion

Reconstruction after excision of malignant tumors of the lower eyelid requires reliable restoration of the posterior lamella to prevent postoperative functional complications. Conventional techniques for posterior lamellar reconstruction, such as the Hughes tarsoconjunctival flap and mucosal grafting, have been widely used; however, these approaches require staged procedures and are associated with temporary occlusion of the visual axis, which remains a notable limitation [4].

In contrast, FTG provides excellent anatomical compatibility by utilizing autologous tarsoconjunctival tissue and allows for single-stage reconstruction. Accordingly, FTG has been widely adopted for posterior lamellar reconstruction in small to moderate defects [5,6]. Nevertheless, because FTG is a free graft that relies entirely on revascularization from the recipient bed, larger grafts are associated with an increased risk of complications, including graft failure, contraction, and postoperative ectropion [7,9]. In addition, comparative surgical series have highlighted both the practicality of FTG as a single-stage option and the need for careful case selection when compared with staged procedures such as the Hughes flap [10]. In the dermatologic series of lower eyelid reconstruction after BCC excision, FTG has therefore been selectively applied with cautious graft/defect size selection [8].

In the present case, FTG was applied to a posterior lamellar defect measuring 15 mm in width, exceeding the size commonly selected in dermatologic practice. Although mild ectropion was observed at the six-month postoperative follow-up, there was no evidence of corneal exposure or lagophthalmos, and the patient reported no subjective symptoms. Overall, favorable functional and cosmetic outcomes were achieved. These findings suggest that FTG may be successfully employed beyond the commonly selected size range when appropriate reconstructive conditions are met. Adequate vascular support has been emphasized as a critical factor for the survival of larger grafts [7]. In the present case, the use of a well-vascularized cheek rotation flap for anterior lamellar reconstruction likely facilitated early revascularization of the FTG, thereby contributing to satisfactory graft survival. This suggests that the combination of FTG with a robust, vascularized anterior lamellar flap may mitigate the risks traditionally associated with larger grafts.

Nevertheless, the findings of this single case do not support the conclusion that FTG is universally applicable to all large posterior lamellar defects. The indication for FTG should be determined through a comprehensive assessment of multiple factors, including the width and height of the defect, the amount of residual tarsus, orbicularis oculi muscle function, and the method used for anterior lamellar reconstruction. Further accumulation of cases is warranted to clarify the relationship between defect size and the risk of postoperative complications and to establish more definitive criteria for patient selection.

Despite these limitations, the present case demonstrates that functional and cosmetic reconstruction can be achieved for relatively large lower eyelid defects while avoiding staged procedures and temporary visual axis occlusion. Accordingly, this case provides clinically relevant evidence supporting the cautious expansion of FTG indications in selected patients undergoing lower eyelid reconstruction, particularly when combined with a robust, well-vascularized anterior lamellar flap.

## Conclusions

This case highlights the practical feasibility of FTG for posterior lamellar reconstruction in a large lower eyelid defect when adequate vascular support is ensured. Although the graft size exceeded that commonly selected in dermatologic practice, a stable postoperative course was achieved without vision-threatening complications. Rather than expanding indications indiscriminately, this case underscores the importance of individualized reconstructive planning, particularly the role of a well-vascularized anterior lamellar flap in supporting graft survival. These findings provide pragmatic insight for clinicians considering single-stage reconstruction in selected lower eyelid defects.
